# The Role of Supplementing a Complex Phytobiotic Feed Additive Containing (*Castanea sativa* mill) Extract in Combination with Calcium Butyrate, Zinc–Methionine and Essential Oils on Growth Indicators, Blood Profile and Carcass Quality of Broiler Chickens

**DOI:** 10.3390/vetsci10030212

**Published:** 2023-03-10

**Authors:** Nikolai P. Buryakov, Artem Yu. Zagarin, Mohamed M. Fathala, Dmitrii E. Aleshin

**Affiliations:** 1 Department of Feeding Animals, Institute of Animal Science and Biology, Russian State Agrarian University—Moscow Timiryazev Agricultural Academy, 49 Timiryazevskaya Str., 127434 Moscow, Russia; n.buryakov@rgau-msha.ru (N.P.B.); azagarin@rgau-msha.ru (A.Y.Z.);; 2 Scientific and Educational Laboratory of Advanced Technologies, Russian State Agrarian University—Moscow Timiryazev Agricultural Academy, 49 Timiryazevskaya Str., 127434 Moscow, Russia; 3 Animal Husbandry and Wealth Development Department, Faculty of Veterinary Medicine, Alexandria University, Alexandria 5424041, Egypt

**Keywords:** phytogenic feed additives, plant extracts, essential oils, Ross 308, growth, carcass and blood parameters

## Abstract

**Simple Summary:**

Environmental microorganisms are always present in poultry farming and may be associated with a high concentration of organic dust, microorganisms in feces, litter, dust and air, as well as the release of volatile odorous compounds, feathers, dandruff (skin material), mites, bacteria, fungi and fungal spores and endotoxins, depending on the type of bird and stages of the production cycle. Moreover, highly productive poultry raised on private farms always have a lower level of immunity compared to backyard poultry flocks. This is due to high productivity, forage stresses during rearing, cohesive plantings and extensive contact between individuals; all these are the result of a decrease in productivity and, possibly, the development of infectious diseases. However, the use of antibacterial drugs for the prevention and treatment of infectious diseases is associated with a number of undesirable risks, the main of which are the contamination of poultry products and the development of resistance of microorganisms to antibiotics. Thus, we assume that plant-based extracts and phytobiotics used as feed additives can be used as alternatives to antibiotics and can definitely affect the productivity, health and product quality of broiler chickens. The complex phytobiotic feed additives (CPFA) contain the following: tannins, eugenol, cinnamon aldehyde, zinc–methionine, calcium butyrate, silicon dioxide and dextrose.

**Abstract:**

The purpose of this study was to determine the level of application and effectiveness of the use of vegetable feed additives from complex phytobiotic feed additives (CPFA) in the diets of broiler chickens, as well as their effects on growth indicators, carcass characters and blood profile. A total of 258 Ross 308 chicks were divided into six dietary regimens, including: a basal diet without additives as a first control group (CON); the second group received a basal diet supplemented with 200 g/t in the starter phase and 100 g/t in the grower and finisher phase; the third group—400 g/t and 200 g/t; the fourth group—600 g/t and 300 g/t; the fifth group—800 g/t and 400 g/t; and the sixth group—1000 g/t and 500 g/t of a complex phytobiotic supplement based on tannins, respectively. The CPFA contains the following: tannins 36.8–55.2%, eugenol 0.4–0.6%, cinnamon aldehyde 0.8–1.2%, zinc–methionine 1.6–2.4%, calcium butyrate 8–12%, silicon dioxide 1.2–1.8% and dextrose up to 100%. The maximum introduction of phytobiotics (1000 g/t) at 7 days of age leads to a decrease in the live weight of broilers which reduced by 8.27% (*p* < 0.05) compared to the minimum level of phytobiotics (200 g/t). From 15–21 days, the live weight was significant between the supplemented and control groups and represented 396.21, 384.81 and 384.16 vs. 316.91 g for the CPFA 4, CPFA 5, CPFA 1 and control group, respectively. Furthermore, the same trend was recorded in the average daily gain during the periods between 15–21 and 22–28 days of the experiment. Feeding CPFA had a positive effect on the carcass indicators, except for the feeding of CPFA 3 in the amount of 600 g/t in the starter phase and 300 g/t in the grower and finish phases, which recorded the lowest weight in relation to the CPFA 1 and 2 groups and represented 1309.58 vs. 1460.06 and 1456.52 g, respectively, and the difference was significant. The inclusion of CPFA in poultry diets contributed to an increase in lung mass in the experimental groups relative to the control group, except for the CPFA 5 group which represented the lowest weight of lung mass (6.51 g) and the differences were significant between the CPFA 2 and CPFA 3 and the control groups. The highest concentration of leukocytes was observed during the experiment period in the group of poultry receiving phytobiotics (CPFA 3), which significantly exceeded the control group by 2.37 × 109/L. A significant decrease in the level of cholesterol was recorded in the CPFA groups when compared to the control group and represented 2.83 vs. 3.55 mmol/L, respectively. Consequently, the introduction of vegetable feed additives from complex phytobiotic feed additives (CPFA) in the diets of Ross 308 chicks had a positive effect on the growth production, the carcass yield, the mass of the pectoral muscles and the mass of the lungs. Moreover, it did not cause a harmful effect on the biochemical parameters of the blood.

## 1. Introduction

In recent decades, there has been a significant growth in the human population on the planet, and according to some sources, the earth’s population will amount to 10 billion people by 2050, which will directly lead to an increased demand for food, especially inexpensive meat products produced from the meat of farm animals, including poultry [1,2,3]. Consequently, an increase in the standard of living and the growth of the world’s population are key factors in the increasing demand for poultry products, which are an affordable source of inexpensive animal protein and meat products [3,4,5].

Poultry farming is characterized as the most intensively developing branch of agriculture in the world. Thus, according to the assessment of the Economic Research Service of the US Department of Agriculture, over the past 20 years, poultry has become the most consumed livestock product in the world, especially in developing countries. An estimated increase of 121% in poultry meat production is expected which is much more significant to 66% of beef and 43% of pork [5,6].

Based on the latest estimates, the world production of broiler chicken meat, which in 1961 was about 7.56 million tons, will increase in 2025 than 139.19 million tons, which is 17.0 kg of meat per person. The most significant increase in poultry meat production is observed in Brazil, Russia, Indonesia, Japan and India [3,5,7]. As the demand for poultry products grows in the markets of developing countries, international imports will continue to grow and increase. It is expected that over the next 10 years, poultry meat will remain the world’s largest imported livestock product [5,7].

To fully meet the increased demand for animal food products, countries have increased their domestic production. At the same time, Brazil, the United States, the European Union and Thailand are major producers and exporters of poultry meat. Simultaneously, it is noted that Brazil will be the world’s leading exporter until 2031; at the same time, there is a decline in production in the United States. The high demand for poultry products is also due to the recognition of poultry products as healthier alternatives to human nutrition, characterized by a higher protein content and a lower lipid content. At the same time, the meat of broiler chickens contains more protein of a high-grade amino acid composition compared to the meat of other types of farm animals [3,4,5,6,7].

An extensive system of poultry farming began to use antibiotics as growth stimulants, as in the basis of veterinary and sanitary measures [8,9,10]. The use of antibiotics as growth stimulants contributed to an increase in the productivity of chickens, but this was also a serious problem due to the developing human resistance when consuming products containing antibiotic residues. So, a number of bacteria have ways to reduce the concentration of the antibiotic in the cell, based on the complete destruction of the antibiotic molecule by enzymes or chemical changes into a safer compound [11,12,13,14].

In the countries of the European Union, 25 thousand deaths per year from antibiotic resistance have been recorded. The emerging phenomenon of antibiotic resistance has become a serious medical and environmental problem of mankind, since the residual amounts of antibiotics accumulate in animal products and also enter the external environment [15]. As a result of the negative consequences of the use of antibiotics and the prohibition of the use of antibacterial drugs as growth stimulants in the European Union (2006) [16,17,18] and the USA (2017) [17,19], their use as a feed additive was prohibited. Currently, manufacturers of feed additives face two tasks: the production of additives with a bactericidal effect, as well as stimulating the growth and development of high-quality muscle tissue [20,21,22,23]. There are more than a dozen solutions on the market having the above properties with varying degrees of effectiveness, such as probiotics [19,22,24], prebiotics [24,25,26], symbiotics [25,26,27], enzymes [25,26], phytobiotics [2,3,13,20,21] and plant extracts [8,9,14,23]. Consequently, the aim of our study was to assess complex phytobiotic feed additives (CPFA) and their combined effect on the productivity, meat performance and blood picture of broiler chickens.

## 2. Materials and Methods

This animal study has been reviewed and approved by the Ethics Committee of the Russian State Agrarian University—Moscow Timiryazev Agricultural Academy (protocol 2022-8 date 6 May 2022).

### 2.1. Experimental Design, Characteristics of Objects and Conditions of Research

The present research was conducted from 29 July 2022 to 2 September 2022 on the basis of the Center of the Safety and Effectiveness of Feed and Additives of the Research Institute for Advanced Research and Innovation in the Agro-industrial Complex of the Volgograd State Agrarian University (Volgograd, Russia). By the method of balanced groups of analogues, taking into account their origin, general development and live weight, six groups of Ross 308 cross broiler chickens were formed. The chickens were purchased at the poultry farm of the KHK JSC Krasnodonskoe of the Ilovlinsky district of the Volgograd region. A total of 258 chicks aged 1 day were randomly divided into six groups. The number in each group was 43 heads (Table 1).

The entire experimental bird was kept in one poultry house. The separation into sections of equal area of 1300 × 1500 cm was carried out using partitions that do not allow the mixing of chickens. The system of keeping chickens is outdoor with the use of deep litter (sawdust). The birds had free access to food and water. The microclimate indicators corresponded to the requirements of the content of the cross. Bunker feeders (one feeder per group) were used to ensure the feeding of the bird; feeding was carried out manually daily.

Nipple drinkers with drop catchers (seven drinkers per group) were used to ensure the watering of the bird. In the course of the experiment, complete compound feeds with the same nutritional value and chemical composition were used in feeding broiler chickens. Thus, the conditions of keeping and feeding, planting density, watering and the feeding front of chickens in all groups were identical and met the requirements of the Ross 308 cross. The scientific experiment included the entire period of broiler chickens growing, which lasted for 35 days.

### 2.2. Diets, Feed Analysis, Broiler Chicken Nutrition and Phytobiotic Feed Additive

Chickens of all groups were fed the same complete compound feeds (Starter—0–10 days; Grower—11–24 days, Finisher 1—25–30 days; and Finisher 2—from 31–35 days), which were balanced in nutrients, metabolic energy and amino acid ratio and corresponded to the recommended nutrition characteristics of broiler chickens of the Ross 308 cross of Aviagen (2022) [28] (Table 2 and Table 3). Feeding was ad libitum. The first group of broiler chickens served as a control and received complete compound feeds (basal diet) during the experiment without the introduction of complex phytobiotic feed additives. The broiler chickens of the experimental groups were fed complete phase compound feeds with the introduction of a phytobiotic feed additive base which contains tannins, calcium butyrate and other auxiliary substances.

The feed additive under study is a powder from pale brown to brown in color; the main active constituents of the used feed additive are the tannins of the *Castanea sativa* Mill, calcium butyrate, zinc–methionine, cinnamon aldehyde and eugenol. The feed additive contains the following (provided by the manufacturer): tannins—36.8–55.2%, eugenol 0.4–0.6%, cinnamon aldehyde 0.8–1.2%, zinc–methionine 1.6–2.4%, calcium butyrate 8–12%, silicon dioxide 1.2–1.8% and dextrose up to 100%.

The studied additive was introduced into the composition of compound feeds as part of a premix by stepwise mixing on the basis of the feed mill of Megamix LLC. Tannins were not introduced into the diet during the second finishing period. Formulas for the complete feed for poultry were developed using the computer program Feed Optima (v. 2020.8.17251), taking into account the chemical composition of the components, which was determined at the scientific testing center Cherkizovo (Moscow, Russia).

### 2.3. Sample Collection and Chemical Analysis

Before the preparation of rations, each raw material was subjected to nutritional value analysis. The moisture content (%) in all samples was determined in accordance with the standard methods of the Association of Official Analytical Chemists. Humidity (2000) [29] was determined by drying the sample at 100–105 °C for 24 h (DM) (method 930.15). After drying, the samples were weighed and subjected to salting at 500 °C for 6 h to determine crude ash (method 942.050).

The content of the ether extract was determined by extraction with petroleum ether (method 920.39). The crude protein content (N × 6.25) (method 976.05) was determined by the Kjeldahl method using an automatic analyzer Kjeldahl, K1100F (Foss electric LLC, DK-3400 Hilleroed, Denmark).

### 2.4. Productivity and Quality Indicators of Carcass in Broiler Chickens

To assess the productivity of broiler chickens, the dynamics of live weight (LW), average daily gain, livestock safety, feed costs per 1 kg of live weight gain, and the production efficiency factor (*PEF*) were taken into account (Formula (1)) [30]. The individual weighing of chickens (*n* = 43) in each group was carried out to determine the dynamics of *live weight* and calculate the average daily and absolute growth of the chickens, starting from the daily age of the birds to weekly (1 day, 7 days, 14 days, 21 days, 28 days and 35 days).

(1) $$PEF=\frac{live\;weight\;\left(\mathrm{kg}\right)\times Viability\left(\%\right)\times 100}{age\;of\;the\;poultry\;\left(\mathrm{days}\right)\times cost\;of\;feed\;per\;1\;\mathrm{kg}\;of\;gain\;\left(\mathrm{kg}\right)}$$

Weighing was carried out using the electronic laboratory scales Mercury 122ACF-3000.05 (MERCURY WP TECH GROUP CO., LTD., Seoul, Republic of Korea). With a discreteness of 0.05 g, the live weight of the chickens was recorded in the forms of individual weighing of broiler chickens.

1. The live weight (g) was determined by the control individual weighing of broiler chickens at the age of 7, 14, 21, 28 and 32 days, and before slaughter.
2. The absolute live weight gain (*AWG*, g) is the increase in live weight over the period of the experiment; it was determined using Equation (2):

(2) $$AWG=W_{2}-W_{1}\;$$

where *W*_2_ is the live weight of the broilers at the end of the growing period (final LW, g) and *W*_1_ is the live weight of the broilers at the beginning of the growing period (initial LW, g).

3. Average daily gain (*ADG*, g)—calculated by the weighing results; determined by Formula (3):

(3) $$ADG=\frac{W_{2}-W_{1}}{t_{2}-t_{1}}\;$$

where *W*_2_ is the live weight of the broilers at the end of the growing period, g; *W*_1_ is the live weight of the broilers at the beginning of the growing period, g; *t*_2_ is the age of the chicks at the end of the growing period, days; and *t*_1_ is the age of the chicks at the beginning of the growing period, days.

4. Feed costs (FC) were calculated based on the data of the feed consumption log and gross body weight gain in each group. Feed costs per 1 kg of live weight gain (kg)—by dividing the amount of feed consumed over the entire period of the experiment by the live weight gain of the broiler chickens during the growing period.
5. The safety of the livestock was calculated as the ratio of livestock at the end of cultivation to livestock at planting, expressed as a percentage.

During the feeding trial, the number of deaths was counted to calculate the survival rate as the percentage of the surviving to the initial number of broilers.

At the age of 35 days, in accordance with the methodology of the All-Russian Scientific Research and Technological Institute of Poultry Farming [30], a controlled slaughter of poultry and anatomical cutting of the broiler chicken carcasses was carried out.

All broiler chickens from each group with a body weight close to the average value for the group were selected for studying carcass quality. The selected chickens were not given food for 10 h before slaughter, while provided with constant access to water. The broilers were killed in accordance with the recommendations by the FOSR for the euthanasia of experimental animals.

In the process of slaughtering, the following parameters of meat productivity were taken into account: the mass of the gutted carcass (g), the mass of the pectoral and femoral muscles, shin muscles and the mass of the internal organs (liver, muscular and glandular stomach, heart, spleen and lungs). Anatomical cutting was carried out on the basis of the testing laboratory analysis of feed and livestock products at Volgograd State Agrarian University.

### 2.5. Blood Sampling and Analysis

To assess the biochemical parameters and the direction of metabolism in the poultry body, we conducted blood sampling in order to study the biochemical (AST, ALT, glucose, cholesterol, triglycerides, calcium, phosphorus, total protein, albumins and globulins) and morphological (hemoglobin, erythrocytes and leukocytes) parameters.

Blood was taken from thirty animals from each group with average live weight at the age of 35 days of life. The blood samples were taken at slaughter by an internal method, cutting the junction of the jugular and bridge veins in the oral cavity.

The samples for the biochemical blood testing were collected in vacuum tubes with a coagulation coagulant (Zhejiang Gongdong Medical Technology Co., Ltd., Huangyan, China). After the test tubes were centrifuged at 3000 rpm for 10 min, the separated serum was moved to sterile Eppendorf-type test tubes. The serum was stored at −20 °C for further analysis. Biochemical blood parameters were determined in a laboratory (Moscow, Russia) using an automatic biochemical and enzyme immunoassay analyzer ChemWell 2910 Combi (Awareness Technology, Inc., Palm City, FL, USA).

Whole blood for research was collected in tubes with K2 EDTA (Guangzhou Improve Medical Instruments Co., Ltd., Huangyan, China). The morphological parameters and hemoglobin were analyzed using the MEK 6450 analyzer (Nihon Kohden, Tokyo, Japan), using AVANTOR control materials. Coloring: by May-Grunwald (Gemstandart, St. Petersburg, Russia); microscopy using Meiji Techno Nikon (Meiji Techno, Iruma-gun Saitama, Japan).

### 2.6. Statistical Analysis

Before carrying out the statistical analysis, all data were tested for normality and homogeneity by Shapiro–Wilk’s and Levene’s tests, respectively. Before processing the percentile data, an arcsine transformation was used [31]. The data were statistically analyzed using the statistical analysis program SPSS, 2017 [32]. A one-way ANOVA followed by Tukey’s multiple comparison tests (post-hoc test) were used to check the significance and compare the experimental groups, according to the following statistical model:

Xijk = μ + Ai + eijk

where Xik = an individual observation; μ = overall mean; Ai = effect of ith treatment; and eik = random error.

## 3. Results

### 3.1. Growth Performance and Meat Quality of Ross 308 Broiler Chickens

The results of the dynamics of the live weight of the Ross 308 cross broiler chickens are given in Table 4.

When starting the study, the initial live body weights of the chickens were nearly identical; the difference in the average values did not exceed 1%. At 7 days of age, the chickens in the CPFA 4 group recorded the highest live body weights, while the chickens of the CPFA 5 group represented the lowest body weights and represented 173.05 vs. 155.40 g for the CPFA 4 and CPFA 5 groups, respectively, and the difference was significant. On the other hand, at the age of 14 days, the differences between the experimental groups were not significant and the highest value was recorded in CPFA 4 (433.79 g), while the lowest was recorded in the control group (417.05 g). Throughout the subsequent intervals (21-, 28- and 35-days-old), the differences between the groups were significant. At 21- and 28-days-old, there was a significant difference between CPFA 4 and the control group and the values represented 840.00 vs. 733.95 g and 1381.86 vs. 1254.42 g, respectively.

The live weight of the chickens in the 2nd, 4th and 5th experimental groups was higher compared to the control group by 3.61%, 4.21 and 5.85%, respectively; however, the chickens fed tannins in the maximum experimental amount had the lowest live weight at the age of 7 days—4.95% less than the live weight of the control chickens group. 

At the age of 14 days, the chickens of all experimental groups outperformed the control group in live weight by 6.96%, 1.34, 2.46, 6.41 and 3.79%, respectively. Moreover, at the age of 21 days, the live weight of the chickens receiving tannins as part of compound feeds exceeded the live weight of the chickens without their inclusion by 13.11%, 2.54, 3.61, 14.45 and 11.41%, respectively. The chickens of the CPFA 3 group did not significantly differ in live weight from the control group at the age of 14 days or at the age of 21 days, while the chickens of the CPFA 4 group recorded a significant difference in live weight when compared to the control group and represented 480.00 vs. 733.95 g at the age of 21 days. At 28 days, the live weight of the chickens in the CPFA 2 and CPFA 3 groups were identical to the values of the control group, while the highest values of live weight were set in the CPFA 4 group of chickens receiving phytobiotics in the amount of 800 g/t in mixed feed for the starter phase and 400 g/t in mixed feed for the grower and finisher phase.

At the end of rearing, the broiler chickens in the CPFA 5 group were distinguished by the highest values of live weight—7.07% higher than in the control group (*p* < 0.05), which amounted to 2007.91 g, while the live weight of the chickens not receiving the additive was 1875.35 g. The chickens in CPFA 4 were also characterized by a higher live weight and represented 1994.65 g, and the difference with the CPFA 2 group, which recorded the lowest final live weight (1849.07 g), was significant. On the other hand, the live weight of the chickens in the CPFA 1 and 3 groups was slightly lower than the control values. The productivity indicators of the broiler chickens are shown in Table 5.

Generally, the absolute live weight gain from the start of the experiment to the end revealed significant differences between the supplemented groups only, while when compared with the control group the differences were *p* > 0.05. Moreover, the same trend was noted in the average daily gain (ADG). During the period from 15–21 days of rearing under the study, the absolute live weight gain recorded a significant difference between the supplemented and control groups and represented 396.21, 384.81 and 384.16 vs. 316.91 g for CPFA4, CPFA5, CPFA1 and the control group, respectively. Furthermore, the same trend was recorded in the average daily gain during the periods between 15–21 and 22–28 days from the start of the experiment. At the age of 35 days, a controlled slaughter and anatomical cutting of broiler chicken carcasses was carried out. The results explaining this are presented in Table 6.

As a result of feeding with a complex phytobiotic feed additive, the weight of the un-eviscerated weight gutted bird carcass had a positive effect on the carcass indicators, except for the feeding of CPFA 3 in the amount of 600 g/t in the starter phase and 300 g/t in the grower and finisher phase, which recorded the lowest weight in relation to the CPFA 1 and 2 groups and represented 1309.58 vs. 1460.06 and 1456.52 g, respectively, and the difference was significant. Regarding the production of pectoral muscles, it should be noted that the mass of the pectoral muscles was characterized by the worst indicators in CPFA 3 chickens, where this indicator was 332.43 vs. 385.96 g against the control group, while the highest weight of pectoral muscles was recorded in the CPFA 1 group which represented 459.19, and the difference between the CPFA 1 and CPFA 3 groups was significant.

The inclusion of CPFA in poultry concentrates contributed to an increase in lung mass in the experimental groups relative to the control group except for the CPFA 5 group, which represented the lowest weight of lung mass (6.51 g) and the differences were significant between the CPFA 2, CPFA 3 and control groups. The use of a phytobiotic dosage in the amount of 200 g/t and 300 g/t (CPFA 2 and CPFA 3) in the grower and finisher phase is 1.48- and 1.54-times the weight of the lungs higher compared to the control. However, in the CPFA 1 group, there was a decrease (*p* < 0.05) in the size of the spleen in chickens receiving a minimum level of phytobiotics in the amounts of 200 and 100 g/t, respectively.

### 3.2. Morphological Blood Parameters of Broiler Chickens

The results of the morphological parameters of blood are illustrated in Table 7.

The concentration of erythrocytes in the blood of the control chickens, CPFA 1, CPFA 4 and CPFA 5 groups was also approximately at the same level; however, in the blood of the chickens of CPFA 2 and 3, the content of erythrocytes was the lowest, yielding slightly to the control by 6.15 and 7.31%. The most significant difference was found in the level of leukocytes. Chickens with the highest live weight at the end of rearing were characterized by a reduced content of leukocytes in the blood (CPFA 4 and CPFA 5), while in the blood of chickens with the lowest weight, the level of leukocytes was higher than the control values by 12.58–25.48% (CPFA 2 and CPFA 3). The highest concentration of leukocytes was observed during the experiment period in the group of poultry receiving phytobiotics (CPFA 3, *p* < 0.05), which significantly exceeded the control group by 2.37 × 10^9^/L, respectively. With reference to the content of hemoglobin, there were no significant differences between the experimental groups, while the highest level was recorded in the CPFA 4 group and represented 126.67 g/L, while the lowest value was recorded in the CPFA 2 group and represented 121.33 g/L.

### 3.3. Biochemical Blood Parameters of Broiler Chickens

The results of a biochemical study of the blood serum of the broiler chickens are presented in Table 8.

The introduction of phytobiotics at different levels did not negatively result in changes in the biochemical parameters of the blood in the different experimental groups. Moreover, all the obtained biochemical indicators were within the reference physiological values. On the same side, none of the biochemical parameters revealed any significant differences between the different experimental groups (*p* > 0.05), except for the cholesterol level, which recorded a significant decrease in the CPFA 1, 2 and 4 groups when compared to the control group of chickens and represented 3.44, 3.11 and 2.83 vs. 3.55 mmol/L, respectively. On the other hand, the other supplemented groups (CPFA 3 and 5) did not reveal significant differences when compared to the control chickens.

## 4. Discussion

### 4.1. Growth Performance and Meat Quality of Ross 308 Broiler Chickens

Modern industrial poultry farming, both urgent and broiler, is widely used all over the world by many approaches or alternative natural feed additives in feed to increase productivity, improve animal welfare and, ultimately, achieve the sustainability of animal husbandry [2,3,7,8,9,10,11,12,13,14,15,16,17,18,19,20,21,22,23,24,25,26,27,33]. Of course, the ban on the use of antibiotics when feeding poultry and other types of productive farm animals has positively affected the increase in alternative sources of biologically active substances, such as pro- and prebiotics [10,11,22,27], enzymes [11,34], symbiotics [27,34], organic acids [35,36,37,38,39], essential oils [10,11,13,20], medicinal herbs [36,40,41], phytobiotics [7,8,9,11,36,42,43] and nutraceutical complexes with different component ratios [20,22,27]. Furthermore, medicinal plants, organic acids and feed additives based on probiotic and prebiotic cultures have been widely studied and recognized as one of the most promising additives for poultry and, thus, have been tested in numerous studies of broiler productivity, sometimes with contradictory results.

Nevertheless, the mode of action and optimal dosages of many plant extracts and essential oils are still unknown [13,20,34]. In our research, we conducted our studies on multicomponent feed additive tannins of chestnut wood from seeds which contain active principles as *Castanea sativa* Mill extract, calcium butyrate, zinc–methionine, cinnamon aldehyde and eugenol.

The study of phytobiotics shows ambiguous indicators of poultry productivity, so some authors make the claim that phytobiotics do not show a significant effect [42,43]. Others, on the contrary, characterize more intensive growth [8,9,14,22,44]. In our studies, an increase in the live weight of chickens of the experimental groups was found relative to diets without the use of phytobiotics, as well as in relation to different levels of its use.

The introduction of phytobiotics at the maximum level (1000 g/t) had a negative effect on the growth of broiler chickens in the fifth group at the age of 7 days in relation to the other experimental groups. It is well known that adult chickens develop resistance to pathogenic bacteria after the native microflora becomes established. This was probably due to the fact that the tannins were fed with nutraceuticals and had a predominantly inhibitory effect on the digestive process, and, consequently, the poultry of this group grew more slowly. In the later growing periods from 8–14 days, it did not affect the growth rate of the poultry in this study (200–500 g/t), which was also observed in several other studies [45,46,47,48], even with very wide dose ranges (200–800 g/t) [34,36,49,50]. In addition, when analyzing poultry productivity in early studies, Zaikina et al. (2022) [9], conducted on Cobb 500 cross broilers, found that the optimal dosage of phytobiotics for up to 10 days of tannin-based rearing was 800 g/t, while the dosage of 500 g/t (*p* < 0.05) proved to be worse than an antibiotic. It is probable that higher dosages of phytobiotics of 100–1000 g/t for up to 7 days tend to manifest themselves more as anti-nutritional substances that prevent the normal colonization of the gastrointestinal tract with normal microflora. The negative effect of higher doses of tannin extracts can be explained by a significant decrease in feed intake, which leads to a decrease in body weight gain, which is consistent with the results of studies [6,7,11,39]. The positive effect of the low doses of chestnut tannins on body weight and FCR was found in young birds. However, the negative effect of tannin supplements on live weight and FCR is mainly due to a higher dosage of tannins in broilers [6,7,10,11]. Subsequently, the growth of the poultry increased significantly with the introduction of higher levels of phytobiotics in the amount of 100 g/t and 400–500 g/t compared to the control group and the difference was significant. However, at the end of the broiler chicken rearing period, the poultry from the groups receiving 400 g/t and 500 g/t of phytogenic feed additive proved to be the best.

Considering the cutting of carcasses, the yield of a half-gutted carcass was significantly affected by the level of use of a phytogenic feed additive. So, the worst in this indicator was a bird that received a growth phase of 300 g/t of phytobiotics before slaughter and 600 g/t of feed additive in the starter phase. It is probable that such a lower value is due to the fact that higher dosages were used at earlier periods of rearing and, accordingly, the bird gained weight more slowly. However, when comparing between groups for this indicator, for the bird receiving the minimum levels of phytobiotic (100 g/t and 200 g/t), the difference was significant. Similar results were obtained for the weight of the pectoral muscles, which were higher when the phytobiotic was administered in an amount of 100 g/t compared with a dosage of 300 g/t from 11 to 30 days of rearing. The content of the individual organs, such as the lungs, in animals receiving 200 and 300 g/t of feed phytobiotic additives was higher by 48.2% and 53.9%, respectively, which probably contributed to a more intensive gas exchange, and, consequently, higher feed consumption.

The organs of hematopoiesis include the liver and the spleen in which the synthesis and decay of blood cells occur. The spleen in birds is a separate small-sized organ, characterized by a variety of shapes. The spleen not only destroys, but also accumulates, shaped blood elements—erythrocytes, leukocytes and platelets. Based on our results, it was found that the spleen of all birds from the supplemented groups were smaller than the control group; in particular, the bird that received the lowest dosages of phytobiotic feed additives throughout the rearing had a significant decrease in its weight compared to the control. The role of the spleen is very significant because it is responsible for the capture and destruction of endotoxins, insoluble components of cellular detritus in burns, injuries and other tissue damage, its cells recognize antigens foreign to this organism and synthesize specific antibodies.

The use of a single substitute or ideal combinations of various alternatives (a complex of phytobiotics and other nutraceuticals) with the proper management and practice of animal husbandry can play a key role in maximizing productivity and maintaining animal productivity [9,51,52,53,54].

According to a recent study by Lillehoj et al. (2018), the use of a mixture of several phytochemicals has a synergistic effect to reduce the negative effects of intestinal infections [55]. According to studies by Lee et al. (2010), the addition of a mixture of Curcuma longa, Capsicum annuum (pepper) and Lentinus edodes to the diet of just-day-old broiler chickens improved the body weight gain in birds infected with E. acervulina compared to birds fed a control diet or a diet containing Capsicum plus Lentinus [56].

The effect of carvacrol, cinnamon aldehyde and capsicum oleoresin on the regulation of gene expression related to immunology, physiology and metabolism was investigated in Kim et al. (2010) in chickens using high-performance microchip analysis [57]. According to the analysis of the data obtained, it should be noted that high levels of CPFA input at the beginning of cultivation (up to 7 days) in the amount of 1000 g/t probably have a negative effect on the control due to the high bacteriostatic activity of the components of sweet chestnut extract. In Furness et al. (2013) [58], it is noted that numerous studies have shown disease prevention or immunostimulant effects of phytochemicals but only a few have investigated the main mechanisms involved. Some phytochemicals inhibit the innate immune response by acting on pathogen pattern recognition receptors or their downstream signaling molecules [58,59].

### 4.2. Morphological Blood Parameters of Broiler Chickens

Blood cells play an essential role in the body of animals; for example, they are responsible for the transport of nutrients, the exchange of gases, provide protection to the body and have the ability to synthesize immune bodies [36,49,50,51,52,59,60,61,62,63,64,65]. So, it was found that the introduction of phytobiotics did not significantly affect the morphological parameters of blood, except for the content of leukocytes. Leukocytes in the third experimental group, which received 600 g/t and 300 g/t of the phytobiotic feed supplement as part of their diet during the starter and grower and finisher phases, were characterized by the highest content in whole blood compared to other animal groups. This phenomenon can probably be positive, since it is due to an increase in the overall immune protective state of the bird’s body. On the other hand, tannins probably act on the liver body in this dosage as anti-nutritional components of the feed. In the future, it is necessary to evaluate the effect of the addition of tannins on the hematopoietic function in broiler chickens for further study of their effect on immunity and hematopoiesis.

### 4.3. Biochemical Blood Parameters of Broiler Chickens

The biochemical parameters of a bird depend on many factors, such as the environment, the conditions of keeping and feeding, as well as the level of stress. Many authors demonstrated that probiotics, probiotic and phytobiotic feed additives can have significant influence. Others, on the contrary, claim that these additives do not significantly affect the biochemical parameters of animals [60,61,62].

The biochemical parameters of the serum show the metabolism of nutrients in the body and emphasize possible changes caused by internal and external factors [63,64,65]. In veterinary practice, tannins from chestnut extract are most often used, since they are significantly less aggressive to the mucous membrane of the gastrointestinal tract than other tannins [49]. In our study, all the results of biochemical blood parameters were within the normal physiological limit.

The serum concentrations of cholesterol and triglycerides are considered indicators of lipid metabolism [65]. In terms of the cholesterol content, all animal groups differed, and, with the exception of the groups CPFA 2 and CPFA 4, were higher than in the control (*p* > 0.05). The data obtained by us are consistent with the data of Basit et al. (2020) [66], whose study showed that on the 21st day of the supplement, flour from Persicaria odorata leaves did not affect the content of triglycerides and cholesterol in blood serum on the 21st day of cultivation. Opposite results were obtained by Vispute et al. (2019) [21], who reported that dill and hemp seeds significantly reduce serum triglyceride levels during the growth phase. Similar results were obtained by Zhang et al. (2017), who studied that the addition of Chinese bay leaf leaves to the diet of chickens significantly reduced the level of triglycerides and cholesterol in the blood serum [54]. Gilani et al. (2018) found that phytobiotics, organic acids and their combinations lead to a significant decrease in cholesterol and triglycerides in the blood serum of broiler chickens [53]. Zeng et al. (2015) considered the role of herbs and their essential oils; there are suggestions that dietary essential oils can improve their cholesterol-lowering properties in digestion [67].

In our studies, it was found that feeding in the initial period of 800 g/t CPFA (group 4) had a negative effect in the growth of live weight up to 7 days of age, probably due to a decrease in the activity of the microbiota of the digestive tract. However, Tekeli et al. (2006) indicate that the inclusion of phytobiotics did not affect cholesterol concentration (*p* > 0.05) and glucose concentration was increased (*p* < 0.05) in Z. officinale, while triglyceride concentration was increased (*p* < 0.05) in Z. officinale and S. Aromaticum [68].

## 5. Conclusions

Based on our results, the introduction of complex phytobiotic feed additives containing *Castanea sativa* mill extract in combination with calcium butyrate, zinc–methionine and essential oils had a positive effect on the live body weights of broilers, increased the mass of lungs and, moreover, did not cause any harmful effects on the blood profile of Ross 308 broiler chickens, while decreasing the level of cholesterol in some supplemented groups. Moreover, we can conclude that the ideal dose of CPFA for the usage in diets of Ross 308 chickens is 800 g/t in the starter phase and 400–500 g/t in the grower and finisher phase in order to increase the meat production and zootechnical characteristics.

## Figures and Tables

**Table 1:** Feeding scheme and research design.

Groups	Poultry Number (*n*)	Broiler Chicken Feeding Program
CON	43	Basic diet (BD) without Complex Phytobiotic Feed Additives (CPFA) *
CPFA 1	43	BD + CPFA 200 g/t in Starter, 100 g/t CPFA Grower and Finisher *
CPFA 2	43	BD + CPFA 400 g/t in Starter, 200 g/t CPFA Grower and Finisher *
CPFA 3	43	BD + CPFA 600 g/t in Starter, 300 g/t CPFA Grower and Finisher *
CPFA 4	43	BD + CPFA 800 g/t in Starter, 400 g/t CPFA, Grower and Finisher *
CPFA 5	43	BD + CPFA 1000 g/t in Starter, 500 g/t CPFA Grower and Finisher *

* from 31st day of rearing until slaughter, all feed additives were removed from the diet.

**Table 2:** The composition of feeds for broiler chickens.

Ingredients	Age of Poultry (Days)			
	0–10 (Starter)	11–24 (Grower)	25–30 (Finisher 1)	31–35 (Finisher 2)
	The Content of Broiler Chickens in the Feed (%)			
Wheat grain	58.16	60.00	60.00	60.00
Corn grain	7.00	7.58	3.87	3.89
Soybean cake	24.62	-	20.36	20.32
Soybean meal	-	9.42	-	-
Sunflower meal	-	5.52	6.12	6.19
Fish meal	6.05	11.64	-	-
Sunflower oil	-	2.88	5.00	4.99
L-lysine sulphate, 70%	0.52	0.48	0.63	0.63
DL-methionine, 99%	0.37	0.28	0.32	0.31
L-threonine, 98,5%	0.19	0.15	0.16	0.16
L-valine, 96.5%	0.11	0.08	0.08	0.08
L-arginine, 98.5%	0.15	0.12	0.05	0.05
L-isoleucine, 90%	0.11	0.13	0.11	0.11
Sodium chloride	0.18	0.07	0.20	0.20
Monocalcium phosphate	1.04	0.23	1.37	1.37
Sodium sulfate anhydrous	0.03	0.14	0.18	0.18
Potassium carbonate	-	0.20	-	-
Choline, 60%	-	0.08	0.04	0.03
Limestone powder	0.47	-	0.51	0.49
Vitamin-trace mineral premixes *	1.00	1.00	1.00	1.00

* Composition of premises (starter phase) Vitamins: A (thousand IU/kg)—1300; D3 (thousand IU/kg)—500; E (mg/kg)—8000; K3 (mg/kg)—320; B1 (mg/kg)—320; B2 (mg/kg)—860; B5 (mg/kg)—1700; B4 (mg/kg)—80,000; B3 (mg/kg)—6000.0; B6 (mg/kg)—540; B12 (mg/kg)—1.7; Bc (mg/kg)—220; H (mg/ kg)—30.0; Trace elements (mg/kg): iron—2000; copper—1600; zinc—11,000; manganese—12,000; iodine—125; selenium—30.0; NPS enzyme—10.0 kg/t; phytase—5.0 kg/t; coccidiostatics—25.0 kg/t; antioxidant—2.0 kg/t; diatomite (filler)—45.0 kg/t; calcium carbonate (filler)—687.20 kg/t. Composition of premises (growth phase) Vitamins: A (thousand IU/kg)—1100; D3 (thousand IU/kg)—450; E (mg/kg)—6500; K3 (mg/kg)—300; B1 (mg/kg)—250; B2 (mg/kg)—650; B5 (mg/kg)—1500; B4 (mg/kg)—80,000; B3 (mg/kg)—5500.0; B6 (mg/kg)—430; B12 (mg/kg)—1.7; Bc (mg/kg)—190.0; H (mg/ kg)—25.0. Trace elements (mg/kg): iron—2000; copper—1600; zinc—11,000; manganese—12,000; iodine—125; selenium—30.0; NPS enzyme—10.0 kg/t; phytase—5.0 kg/t; coccidiostatics—25.0 kg/t; antioxidant—2.0 kg/t; diatomite (filler)—45.0 kg/t; calcium carbonate (filler)—675.74 kg/t. Composition of premises (finisher 1 phase) Vitamins: A (thousand IU/kg)—1000; D3 (thousand IU/kg)—400; E (mg/kg)—5500; K3 (mg/kg)—220; B1 (mg/kg)—220; B2 (mg/kg)—540; B5 (mg/kg)—1300; B4 (mg/kg)—70,000; B3 (mg/kg)—40,000; B6 (mg/kg)—320; B12 (mg/kg)—1.1; Bc (mg/kg)—160.0; H (mg/ kg)—20.0. Trace elements (mg/kg): iron—2000; copper—1600; zinc—11,000; manganese—12,000; iodine—125; selenium—30.0; NPS enzyme—10.0 kg/t; phytase—5.0 kg/t; coccidiostatics—25.0 kg/t; antioxidant—2.0 kg/t; diatomite (filler)—40.0 kg/t; calcium carbonate (filler)—712.68 kg/t. Composition of premises (finisher 2 phase) Vitamins: A (thousand IU/kg)—1000; D3 (thousand IU/kg)—400; E (mg/kg)—5500; K3 (mg/kg)—220; B1 (mg/kg)—220; B2 (mg/kg)—540; B5 (mg/kg)—1300; B4 (mg/kg)—70,000; B3 (mg/kg)—40,000; B6 (mg/kg)—320; B12 (mg/kg)—1.1; Bc (mg/kg)—160.0; H (mg/ kg)—20.0. Trace elements (mg/kg): iron—2000; copper—1600; zinc—11,000; manganese—12,000; iodine—125; selenium—30.0; NPS enzyme—10.0 kg/t; phytase—5.0 kg/t; antioxidant—2.0 kg/t; diatomite (filler)—40.0 kg/t; calcium carbonate (filler)—737.68 kg/t.

**Table 3:** Nutritional value of compound feeds for broiler chickens.

Nutrients	Age of Poultry (Days)			
	0–10 (Starter)	11–24 (Grower)	25–30 (Finisher 1)	31–35 (Finisher 2)
	Nutritional Value (%)			
Metabolic energy (ME) (kcal/100 g)	301	311	320	320
Crude protein	23.00	21.50	19.04	19.04
Assimilable lysine	1.44	1.29	1.16	1.16
Assimilable methionine	0.72	0.67	0.59	0.58
Assimilable methionine + cystine	1.08	0.99	0.91	0.91
Assimilable threonine	0.97	0.88	0.78	0.78
Assimilable tryptophan	0.28	0.25	0.24	0.24
Assimilable arginine	1.52	1.37	1.22	1.22
Assimilable isoleucine	0.97	0.89	0.81	0.81
Assimilable leucine	1.58	1.42	1.27	1.27
Assimilable valine	1.10	1.00	0.90	0.90
Assimilable histidine	0.54	0.49	0.45	0.45
Crude fiber	3.15	3.39	4.06	4.07
Essential extract	4.85	6.00	8.90	8.89
Linoleic acid	2.20	2.91	5.12	5.11
Linolenic acid	0.14	0.06	0.13	0.13
Calcium	0.96	0.87	0.79	0.79
Assimilable phosphorus	0.48	0.44	0.40	0.40
Magnesium	0.13	0.13	0.15	0.15
Potassium	0.82	0.74	0.77	0.77
Sodium	0.16	0.20	0.16	0.16
Chlorine	0.21	0.21	0.18	0.18

**Table 4:** Dynamics of live weight of broiler chickens, g.

Age of Poultry	CON	Group					*p*-Value
		CPFA 1	CPFA 2	CPFA 3	CPFA 4	CPFA 5	
1 day (initial)	41.72 ± 0.44	41.65 ± 0.50	41.63 ± 0.49	42.05 ± 0.41	41.60 ± 0.45	42.05 ± 0.48	0.962
7 days	163.49 ± 3.27 ^b^	169.40 ± 2.87 ^a^	163.91 ± 2.78 ^ab^	170.37 ± 3.44 ^a^	173.05 ± 3.36 ^a^	155.40 ± 2.32 ^b^	0.001
14 days	417.05 ± 8.40	446.07 ± 7.12	422.63 ± 7.31	427.33 ± 6.89	443.79 ± 9.05	432.86 ± 7.51	0.052
21 days	733.95 ± 13.35 ^c^	830.23 ± 14.11 ^a^	752.56 ± 13.82 ^c^	760.47 ± 11.91 ^bc^	840.00 ± 18.06 ^a^	817.67 ± 15.14 ^ab^	0.001
28 days	1254.42 ± 20.53 ^c^	1380.00 ± 25.73 ^a^	1251.16 ± 23.26 ^c^	1256.74 ± 21.96 ^bc^	1381.86 ± 26.45 ^a^	1353.95 ± 26.22 ^ab^	0.001
35 days (final)	1875.35 ± 31.27 ^bc^	1955.81 ± 34.11 ^abc^	1849.07 ± 35.29 ^c^	1860.70 ± 33.52 ^bc^	1994.65 ± 35.08 ^b^	2007.91 ± 43.22 ^a^	0.002

Values are expressed as means ± standard error. Means denoted within the same row with different superscripts are significant (*p* < 0.05).

**Table 5:** Productivity indicators of Ross 308 cross broiler chickens.

Parameters	Groups						*p*-Value
	CON	CPFA 1	CPFA 2	CPFA 3	CPFA 4	CPFA 5	
AWG, g	1833.63 ± 31.16 ^abc^	1914.16 ± 34.09 ^abc^	1807.44 ± 35.29 ^c^	1818.65 ± 33.62 ^bc^	1953.05 ± 35.01 ^ab^	1965.86 ± 43.31 ^a^	0.002
1–7 days	121.77 ± 3.33 ^ab^	127.74 ± 2.99 ^a^	122.28 ± 2.80 ^ab^	128.33 ± 3.56 ^a^	131.44 ± 3.37 ^a^	113.35 ± 2.38 ^b^	0.001
8–14 days	253.56 ± 9.04	276.67 ± 7.45	258.72 ± 8.23	256.95 ± 8.19	270.74 ± 9.58	277.47 ± 7.59	0.165
15–21 days	316.91 ± 12.91 ^b^	384.16 ± 15.25 ^a^	329.93 ± 15.78 ^ab^	333.14 ± 15.08 ^ab^	396.21 ± 21.56 ^a^	384.81 ± 16.80 ^a^	0.001
22–28 days	520.47 ± 23.42	549.77 ± 29.70	498.60 ± 22.74	496.28 ± 25.95	541.86 ± 33.57	536.28 ± 32.81	0.672
29–35 days	638.57 ± 34.07	575.81 ± 41.95	615.00 ± 37.71	603.95 ± 42.20	628.81 ± 43.18	653.95 ± 47.40	0.814
ADG, g	53.93 ± 0.92 ^abc^	56.30 ± 1.00 ^abc^	53.16 ± 1.04 ^c^	53.49 ± 0.99 ^bc^	57.44 ± 1.03 ^ab^	57.82 ± 1.27 ^a^	0.002
1–7 days	17.39 ± 0.48 ^ab^	18.25 ± 0.43 ^a^	17.47 ± 0.40 ^ab^	18.33 ± 0.51 ^a^	18.78 ± 48 ^a^	16.19 ± 0.34 ^b^	0.001
8–14 days	36.22 ± 1.29	39.52 ± 1.06	36.96 ± 1.18	36.71 ± 1.17	38.68 ± 1.37	39.64 ± 1.08	0.165
15–21 days	45.27 ± 1.84 ^b^	54.88 ± 2.18 ^a^	47.13 ± 2.25 ^ab^	47.59 ± 2.15 ^ab^	56.60 ± 3.08 ^a^	54.97 ± 2.40 ^a^	0.001
22–28 days	45.27 ± 1.84 ^b^	54.88 ± 2.18 ^b^	47.13 ± 2.25 ^b^	47.59 ± 2.15 ^b^	77.41 ± 4.80 ^a^	76.61 ± 4.69 ^a^	0.000
29–35 days	91.22 ± 4.87	82.26 ± 5.99	87.86 ± 5.39	86.28 ± 6.03	89.83 ± 6.17	93.42 ± 6.77	0.814
PEF, U	307.59	335.01	296.14	300.02	348.35	350.02	n/o
Safety, %	100	100	100	100	100	100	n/o
FC, kg	1.742	1.668	1.784	1.772	1.636	1.639	n/o

Values are expressed as means. Means denoted within the same row with different superscripts are significant (*p* < 0.05). n/o—not applicable. AWG—absolute live weight gain; FC—feed costs; PEF—production efficiency factor.

**Table 6:** Carcass indicators and weight of internal organs of broiler chickens.

Parameters	Groups						*p*-Value
	CON	CPFA 1	CPFA 2	CPFA 3	CPFA 4	CPFA 5	
Un-eviscerated weight (g)	1373.24 ± 18.27 ^ab^	1460.06 ± 18.43 ^a^	1456.52 ± 2.97 ^a^	1309.58 ± 18.21 ^b^	1434.84 ± 19.85 ^ab^	1401.69 ± 54.04 ^ab^	0.014
Pectoral muscles (g)	385.96 ± 11.70 ^ab^	459.19 ± 9.64 ^a^	386.63 ± 11.66 ^ab^	332.43 ± 36.00 ^b^	417.28 ± 6.39 ^ab^	449.18 ± 20.56 ^a^	0.005
Thigh muscles (g)	190.26 ± 2.30	168.22 ± 4.20	184.45 ± 16.23	157.38 ± 17.71	132.61 ± 7.78	156.13 ± 26.20	0.156
Leg muscle (g)	136.45 ± 8.74	133.95 ± 3.95	120.83 ± 5.42	119.60 ± 6.57	132.31 ± 5.53	120.08 ± 14.04	0.491
Lungs (g)	7.11 ± 1.13 ^b^	8.91 ± 1.17 ^ab^	10.54 ± 0.26 ^a^	10.94 ± 1.61 ^a^	8.25 ± 0.07 ^ab^	6.51 ± 0.25 ^b^	0.033
Heart (g)	8.29 ± 0.09	6.98 ± 0.61	8.05 ± 0.31	6.99 ± 0.95	6.10 ± 0.50	7.25 ± 0.15	0.108
Hepatic (g)	40.11 ± 2.19	38.59 ± 1.11	43.22 ± 1.50	38.70 ± 1.78	44.03 ± 0.92	39.17 ± 0.32	0.069
Spleen (g)	2.41 ± 0.12 ^a^	1.31 ± 0.04 ^b^	2.18 ± 0.28 ^ab^	1.83 ± 0.21 ^ab^	2.04 ± 0.08 ^ab^	1.93 ± 0.14 ^ab^	0.010
Muscular stomach (g)	20.43 ± 1.77	18.83 ± 1.99	22.05 ± 1.30	22.45 ± 1.23	18.68 ± 1.59	20.29 ± 1.74	0.493
Glandular stomach (g)	6.59 ± 0.29	7.60 ± 1.56	8.51 ± 0.53	7.14 ± 0.05	7.07 ± 0.92	7.34 ± 0.10	0.641

Values are expressed as means ± standard error. Means denoted within the same row with different superscripts are significant (*p* < 0.05).

**Table 7:** Morphological blood parameters of broiler chickens.

Parameters	Groups						*p*-Value
	CON	CPFA 1	CPFA 2	CPFA 3	CPFA 4	CPFA 5	
Erythrocytes, 10^12^/L	2.60 ± 0.08	2.59 ± 0.04	2.44 ± 0.06	2.41 ± 0.03	2.62 ± 0.03	2.52 ± 0.14	0.264
Leukocytes, 10^9^/L	9.30 ± 0.57 ^bc^	8.73 ± 0.33 ^bc^	10.47 ± 0.71 ^ab^	11.67 ± 0.62 ^a^	7.90 ± 0.21 ^c^	7.73 ± 0.03 ^c^	0.001
Hemoglobin, g/L	125.67 ± 1.76	123.67 ± 0.88	121.33 ± 1.86	123.00 ± 2.65	126.67 ± 1.76	125.33 ± 4.81	0.719

Values are expressed as means ± standard error. Means denoted within the same row with different superscripts are significant (*p* < 0.05).

**Table 8:** Biochemical parameters of Ross 308 cross broiler chickens.

Parameters	CON	Groups					*p*-Value
		CPFA 1	CPFA 2	CPFA 3	CPFA 4	CPFA 5	
Glucose, mmol/L	11.67 ± 1.15	12.07 ± 0.35	13.67 ± 0.74	12.77 ± 0.44	11.20 ± 1.08	13.27 ± 0.28	0.234
Total protein, g/L	34.10 ± 1.95	31.60 ± 0.29	32.37 ± 0.32	32.70 ± 1.01	35.90 ± 5.27	32.73 ± 0.45	0.819
Albumin, g/L	12.47 ± 0.41	12.17 ± 0.15	11.87 ± 0.07	11.83 ± 0.48	11.73 ± 0.98	12.53 ± 0.32	0.791
Globulin, g/L	21.63 ± 1.58	19.43 ± 0.23	20.50 ± 0.36	20.87 ± 0.54	24.17 ± 4.28	20.20 ± 0.30	0.591
AST, U/L	285.00 ± 24.09	345.67 ± 50.26	293.33 ± 16.46	302.00 ± 16.44	327.67 ± 32.92	372.00 ± 86.52	0.731
ALT, U/L	1.67 ± 0.33	2.00 ± 0.58	1.67 ± 0.33	2.00 ± 0.00	4.00 ± 1.53	1.67 ± 0.33	0.217
Cholesterol, mmol/L	3.55 ± 0.11 ^a^	3.44 ± 0.32 ^ab^	3.11 ± 0.23 ^ab^	3.57 ± 0.10 ^a^	2.83 ± 0.14 ^b^	3.58 ± 0.20 ^a^	0.023
Triglycerides, mmol/L	0.42 ± 0.04	0.45 ± 0.03	0.57 ± 0.10	0.53 ± 0.03	0.48 ± 0.07	0.51 ± 0.05	0.526
Total calcium, mmol/L	2.83 ± 0.13	2.89 ± 0.11	2.94 ± 0.16	2.92 ± 0.03	2.89 ± 0.20	3.07 ± 0.27	0.942
Phosphorus, mmol/L	2.46 ± 0.20	2.33 ± 0.17	2.33 ± 0.17	2.24 ± 0.10	2.55 ± 0.28	2.13 ± 0.01	0.611

Values are expressed as means ± standard error. AST—aspartate aminotransferase. ALT—alanine aminotransferase. Means denoted within the same row with different superscripts are significant (*p* < 0.05).

## Data Availability

The raw data supporting the conclusions of this article will be made available by the authors without undue reservations. The data presented in this study are available upon request from the corresponding author.
